# Lactate Dehydrogenases as Metabolic Links between Tumor and Stroma in the Tumor Microenvironment

**DOI:** 10.3390/cancers11060750

**Published:** 2019-05-29

**Authors:** Deepshikha Mishra, Debabrata Banerjee

**Affiliations:** Department of Pharmacology, Robert Wood Johnson Medical School, Rutgers, The State University of New Jersey, 675 Hoes Lane West, Research Towers, Room 561, Piscataway, NJ 08854, USA

**Keywords:** lactate dehydrogenase, *LDHA*, *LDHB*, isoenzymes, metabolism, tumor microenvironment, metabolic cooperation, tumor stroma, lactate, combination therapy

## Abstract

Cancer is a metabolic disease in which abnormally proliferating cancer cells rewire metabolic pathways in the tumor microenvironment (TME). Molecular reprogramming in the TME helps cancer cells to fulfill elevated metabolic demands for bioenergetics and cellular biosynthesis. One of the ways through which cancer cell achieve this is by regulating the expression of metabolic enzymes. Lactate dehydrogenase (LDH) is the primary metabolic enzyme that converts pyruvate to lactate and vice versa. LDH also plays a significant role in regulating nutrient exchange between tumor and stroma. Thus, targeting human lactate dehydrogenase for treating advanced carcinomas may be of benefit. LDHA and LDHB, two isoenzymes of LDH, participate in tumor stroma metabolic interaction and exchange of metabolic fuel and thus could serve as potential anticancer drug targets. This article reviews recent research discussing the roles of lactate dehydrogenase in cancer metabolism. As molecular regulation of *LDHA* and *LDHB* in different cancer remains obscure, we also review signaling pathways regulating *LDHA* and *LDHB* expression. We highlight on the role of small molecule inhibitors in targeting LDH activity and we emphasize the development of safer and more effective LDH inhibitors. We trust that this review will also generate interest in designing combination therapies based on LDH inhibition, with *LDHA* being targeted in tumors and *LDHB* in stromal cells for better treatment outcome.

## 1. Introduction

The tumor microenvironment (TME) is a complex dynamic cellular environment comprised of tumor cells, stromal cells, blood vessels, extracellular matrix (ECM), growth factors and cellular metabolites [1]. Constant crosstalk between the components and cancer cells reprograms the tumor microenvironment and helps cancer cells to meet their high metabolic demands and impact overall tumor growth [2]. A characteristic feature of tumor microenvironment is deregulated metabolic properties. Cancer cells establish increased metabolic interactions by utilizing opportunistic modes for nutrient acquisition by differential uptake of glucose and amino acids [3] as well as increase in lipids, proteins and nucleic acid biosynthesis for growth [4]. Cancer cells preferentially convert glucose to lactate by the process of aerobic glycolysis also termed the “Warburg effect” [5]. Tumor mass, often a partially independent entity, reprograms sources of metabolite supply to flourish in an otherwise nutrient depleted microenvironment. [2]. If not all, a wide variety of cancers even under normoxic conditions display accelerated glycolysis. The “Warburg” lactate produced and extruded into the microenvironment acts as an alternative metabolic substrate for oxygenated tumor cells in the TME. This adaption of preferential utilization of lactate by oxygenated tumor cells benefits the neighboring hypoxic tumor cells that now can utilize the spared glucose [6].

Lactate dehydrogenase (LDH) is one of the key metabolic enzymes present in the TME that play essential role in conversion of pyruvate to lactate and vice versa making it an important player in cancer metabolism [7]. Deregulated levels of LDHs have been previously reported in multiple tumors, including pancreatic cancer [8,9,10], breast cancer [11], nasopharyngeal cancer [12], gastric cancer [13], bladder cancer [14] and endometrial cancer [15].

In this review, we highlight the roles of LDH and its clinical relevance in the tumor microenvironment. We summarize the molecular regulation of *LDHA* and *LDHB* and emphasize on the importance of lactate; a metabolic substrate of LDH as an additional metabolic energy source and its diverse role in the TME. We show that targeting *LDHA* and *LDHB* expression eventually enhances the cytotoxicity of conventional chemotherapeutic drugs through sensitization. This review highlights the advantage of using complimentary therapies based upon targeting metabolic enzymes in the TME for better outcomes.

## 2. Lactate Dehydrogenase

Lactate dehydrogenase (EC1.1.1.27; L-lactate: nicotinamide adenine dinucleotide [NAD^+^] oxidoreductase), is a tetrameric enzyme of the glycolytic pathway belonging to the 2-hydroxyacid oxidoreductase family. It catalyzes the simultaneous, reversible conversion of pyruvate to lactate with regeneration of NADH to NAD^+^ by 14 orders of magnitude [7]. LDH is composed of two different subunits LDHA (M) and LDHB (H), encoded in human by *LDHA* and *LDHB* genes located on chromosome 11p15.1 and 12p12.1. The two subunits can combine in five different combinations to form homo- or hetero- tetramers in human tissues. Isoenzyme associations formed are: LDH-1 (4H), LDH-2 (3H1M), LDH-3 (2H2M), LDH-4 (1H3M) and LDH-5 (4M). LDH is localized intracellularly, and shows varied isoenzyme composition among the different tissues. LDHA isoform is expressed mainly in skeletal muscles and preferentially converts pyruvate to lactate, while, LDHB isoform is expressed mainly in heart and brain and preferentially converts lactate to pyruvate [16].

**Figure 1:**
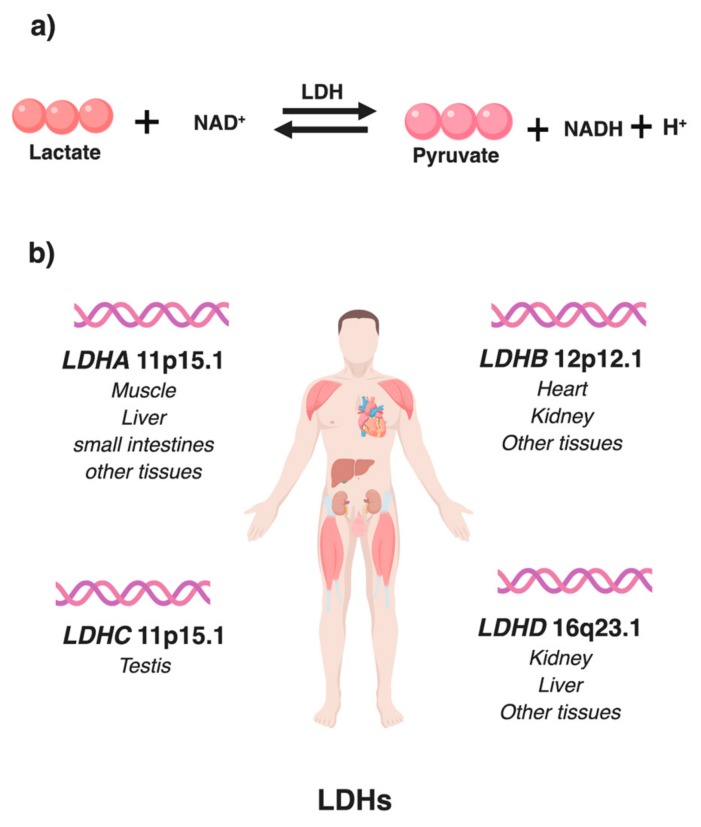
Lactate dehydrogenase: (**a**) Reversible conversion of pyruvate and NADH to lactate and NAD+ catalyzed by lactate dehydrogenase (LDH); (**b**) Human LDH genes showing their chromosomal location and sites of predominant tissue specific expression.

LDHs are highly conserved and believed to arise by gene duplication [16]. As shown in Figure 1, four LDH genes, *LDHA, LDHB, LDHC* and *LDHD* have been described in vertebrates. First three of them utilize L-lactate [7], whereas the fourth, LDHD, utilizes D-Lactate [17,18]. *LDHC*, also known as *LDHX* is a testis-specific gene [16]. In the last few years, some studies have explored its role in cancer and found that in breast cancer cells *LDHC* promotes tumor invasion and migration [19]. Another study on renal cell carcinoma patient samples revealed that *LDHC* expression level were significantly upregulated in cancer tissues and positively correlated with shorter progression-free survival [20]. Role of LDHC and LDHD has not been fully explored in cancers; a recent loss-of-function study in *LDHD* identified two different homozygous variants of *LDHD* resulting in enzymatic loss-of-function and increased concentrations of D-lactate. This is the first study to illustrate that LDHD plays the primary role in human D-lactate metabolism [21]. Another study in clear cell renal cell carcinoma (ccRCC), patients revealed that low *LDHD* expression was a significant predictor of poor prognosis and was associated with poor overall survival [22]. 

A recent report from Ždralević et al., highlighted that deletion of both *LDHA* and *LDHB* is necessary to suppress fermentative glycolysis as disruption of LDH activity by individual *LDHA* and *LDHB* gene knockout failed to reduce lactate secretion whereas, LDHA/B-DKO (double knockout) fully suppressed LDH activity and lactate secretion. Under normoxic conditions, the LDHA/B-DKO cells retained the ability to survive by shifting their metabolism to oxidative phosphorylation (OXPHOS) with reduced cell proliferation; under hypoxic conditions, LDHA/B suppression completely terminated in vitro growth. [23]. 

Despite having significant structural similarities, LDH isoenzymes exhibit distinct kinetic profiles due to differences in charged residues surrounding the active sites [24]. Differences in LDH isoenzyme active site geometries following binding with different ligands such as L-lactate, pyruvate or oxamate, suggests differences in binding affinities and energies [25,26]. A difference in net charge on the molecule guides choice of ligand with higher affinity; LDHA with a negative 6 (−6) net charge has higher affinity for pyruvate as compared to a net positive charge (+1) of LDHB which has higher affinity for lactate [24]. Under normal physiological conditions, each tissue has its own specific LDH expression profile which is controlled at multiple transcriptional and post translational levels, as discussed in later sections (Section 3 and Section 4).

In cancer cells even in the presence of oxygen a major portion of pyruvate generated from glycolysis is directed away from the mitochondria to generate lactate with the help of LDH (the Warburg effect). The generation of different precursor molecules by this process is beneficial for the proliferation of cancer cells [27]. The lactate and proton present in the tumor milieu together protect cancer cells from glucose deprivation by regulating metabolic phenotype of cancer cells [28]. As different tumors have different energy requirements and metabolic rates, they exhibit significant heterogeneity. A variety of factors present in the tumor microenvironment such as hypoxia, pH and intracellular signaling pathways also regulate tumor cell metabolism [29]. The intracellular pathways may become oncogenic and drive metabolic reprogramming by generating enhanced quantities of precursors for synthesis of biomolecules enabling cell growth and proliferation [30]. Therapeutic strategies designed to target metabolic enzymes may improve treatment outcomes. Targeting *LDHA* and *LDHB* may help in arresting the growth of tumors by shutting down the complementary fuel supply altogether. Different signaling pathways affect regulation of LDHs and contribute to altered metabolic phenotypes of cancer cells [8,9,12,13,14,15,31].

## 3. *LDHA* in Cancers

In humans, the tetrameric isoenzyme LDH-5 is predominantly found in muscle tissues and is encoded by *LDHA* gene located on chromosome location 11p15.1. In cancer cells, LDHA helps in rapid conversion of pyruvate to lactate, minimizing pyruvate entry into TCA cycle in the mitochondria. High levels of LDHA helps cancer cells to establish and proliferate by promoting epithelial to mesenchymal transition [31], angiogenesis [15], cytoskeletal remodeling [32], increasing cell motility [33], invasion and migration [34]. One advantage of LDH-5 upregulation for cancer cells is to maintain fuel supply under hypoxic conditions and expectedly a broad range of highly invasive hypoxic cancers show high LDH-5 levels. Table 1 shows some of mechanisms behind *LDHA*- mediated tumor growth.

### 3.1. HIF-1 and LDHA

Oxygen concentrations below 21% (normoxia) is called hypoxia and is graded as: physiological hypoxia: 2–9%; mild hypoxia: 1–5%; hypoxia: <1% and anoxia: <0.1% O_2_ [35] and leads to the activation of a set of transcription factors known as hypoxia-inducible factors (HIFs) [36]. Hypoxia-inducible factor 1 (HIF-1) is a transcription factor composed of two subunits, HIF-1α and HIF-1β, and is sensitive to low levels of oxygen in the microenvironment. In the last few years it has already been established that HIF-1 plays significant role in different cancers. HIF-1 regulates transcription of genes encoding glycolytic pathway enzymes, one of them being LDHA which exhibits oxygen dependent regulation [37]; promoter analysis of *LDHA* revealed the presence of 2 functionally essential HIF-1 transcription factor binding sites within the hypoxia response elements (HREs) that are recognized by HIF-1 [38]. Patients with poor prognosis show LDH-5 overexpression in tissues and linked to tumor hypoxia, increased angiogenic factor production [39,40] and also exhibit strong correlation with VEGF (another target of HIF regulation) expression [40]. 

### 3.2. c-Myc and LDHA

*LDHA* is a c-Myc-responsive gene and its overexpression is necessary for c-Myc-mediated transformation [41]. Furthermore, it has been found that *LDHA* interacts with another c-Myc target gene Rcl that is known to induce anchorage-independent growth. *LDHA* partners with Rcl and acts synergistically to induce anchorage-independent growth establishing a role of *LDHA* in cancer progression [42]. It has also been found that in glucose deprived conditions, c-Myc-transformed fibroblasts and cancer cells undergo extensive apoptosis through a unique glucose-dependent apoptotic pathway involving LDHA [43]. 

### 3.3. FOXM1 and LDHA

Forkhead box protein M1 (FOXM1) is another important transcription factor that plays an important role in cancer development and progression. In pancreatic tumors and cancer cell lines, *FOXM1* and *LDHA* were found to be overexpressed; increased expression of FOXM1 upregulated LDHA activity and expression at both mRNA and protein level. Further investigation revealed that FOXM1 binds directly to the *LDHA* promoter region and regulates its expression [8]. Similarly, in gastric carcinoma (GC) LDHA overexpression is transcriptionally regulated by FOXM1; overexpressed LDHA resulted in a glycolytic phenotype of cancer cells and promoted GC progression [13]. 

### 3.4. KLF4 and LDHA

Krüppel-like factor 4 (KLF4), a member of the zinc finger transcription factor family inhibits cancer EMT and metastasis via transcriptionally downregulating CAV-1 expression by binding directly to the promoter region of the gene [44]. A negative correlation was reported in between *KLF4* and *LDHA* expression; *KLF4* under expression and *LDHA* overexpression were clinically correlated with disease stage and tumor differentiation in patient samples. In vitro KLF4 overexpression led to significant attenuation of the aerobic glycolysis and inhibited growth in pancreatic cancer cells. Mechanistically, it was found that KLF4 negatively regulates transcription activity by binding directly to the promoter regions of the *LDHA* gene [9]. 

### 3.5. Other Pathways

LDHA phosphorylation also contributes to tumor metastasis via altering cell metabolism. It has been shown that human epidermal growth factor receptor 2 (HER2) and avian sarcoma viral oncogene v-src homolog (SRC) activate LDHA through phosphorylation at tyrosine 10 residue which further results in pro-invasive and pro-metastatic behavior of cancer cells [45]. LDH-5 overexpression has also been reported to be significantly associated with phosphorylated VEGFR2/KDR receptor expression [15]. In cancer cells LDHA is tyrosine phosphorylated [46] and was found to be phosphorylated at all four tyrosine sites by fibroblast growth factor receptor 1 (FGFR1). Differential regulation of LDHA and LDHB by FGFR1 tyrosine kinase results in increased stability of LDHA by tyrosine phosphorylation and reduced LDHB expression by promoter methylation; eventually shifting cell metabolism from oxidative phosphorylation to aerobic glycolysis type [47].

Histone demethylases such as Jumonji C domain 2A (JMJD2A), also plays a vital role in cancer development and progression. In nasopharyngeal carcinoma (NPC), JMJD2A promotes warburg effect by transactivating LDHA expression. JMJD2A regulates LDHA expression by binding to LDHA promoter region and activated JMJD2A-LDHA signaling pathway promotes NPC progression [12]. Epithelial-mesenchymal transition (EMT) is a requirement for the progression and metastasis of cancer cells and is very often regulated by acquisition of cancer stem cell (CSC) properties. One of the other ways through which LDHA promotes tumor progression is by regulating cancer stem cell markers [11] and a positive correlation between LDHA expression and CSC/EMT markers has been noted. LDHA promotes cancer progression by regulating epithelial-to-mesenchymal transition (EMT) related genes such as Snail, Slug, E cadherin, N-cadherin, Fibronectin, Vimentin and by upregulating expression of stemness related genes such as OCT4, SOX2, Nanog and c-Myc [31]. 

## 4. *LDHB* in Cancers

Lactate dehydrogenase B (*LDHB*) gene encodes LDH-1. *LDHB* expression in different cancers may also serve as a predictive metabolic marker for therapy response. In breast cancers, *LDHB* expression was found to be a marker for neoadjuvant chemotherapy response evaluation. Breast cancer cell lines with glycolytic and basal-like phenotypes expressed high *LDHB* levels and stable knockdown of *LDHB* reduced glycolytic dependence. Patients with basal-like cancers expressed high LDHB and presented pathological complete response (pCR) to neoadjuvant chemotherapy [48]. On the other hand, elevated LDHB protein levels in oral squamous cell carcinoma (OSCC) patients were found to be associated with poor response to neoadjuvant chemotherapy, high glycolytic dependence and poor treatment outcomes. Overall, high LDHB levels in OSCC present with poor overall survival as well as disease-free survival and resistance to taxol; *LDHB* deletion sensitized OSCC cell lines to taxol and induced cell apoptosis [49]. In osteosarcoma cell lines, *LDHB* was found to be highly expressed with an elevated mRNA levels in tissues with metastasis; advanced stages and recurrence and was overall associated with a poor prognosis in osteosarcoma patients [50]. 

### 4.1. LDHB Promoter Hypermethylation

Different cancers such as prostate, breast (ductal carcinoma in situ) and pancreatic cancers show suppressed or loss of *LDHB* expression as a crucial and early event in cancer development and progression [51,52,53,54,55]. Loss of *LDHB* expression due to promoter hypermethylation was found to be significantly associated with metastatic progression [51,56]. It was found that *LDHB* promoter hypermethylation was not a genomic alteration but an epigenetic abnormality [52]. Suppressed expression of *LDHB* led to a glycolytic transition and revealed the role of suppressed *LDHB* in promoting proliferation, invasion, and migration of cancer cells under hypoxic condition [53]. A lower expression [57] or total loss [58] of *LDHB* was correlated with unfavorable survival outcomes in patient samples. Exact mechanism through which LDHB contributes to cancer development and progression is not fully understood but changes in *LDHB* expression are often associated with early metabolic adaptations [11]. 

### 4.2. LDHB Regulation by Different Pathways

LDHB is also regulated in an mTOR-dependent manner and was found to be transactivated by STAT3 a downstream mTOR effector critical for mTOR-mediated tumorigenesis [59]. Cancer cells demonstrate metabolic adaptability and in a scenario of glucose deprivation, LDHB helps in sustaining autophagy via lactate. Consuming lactate instead of glucose mimics a glucose deprived condition for cancer cells and promotes autophagy. LDHB plays a major role in metabolic adaptability of cancer cells by controlling lysosomal activity and autophagy enabling oxidative phenotype cancer cells to use lactate preferentially over glucose, leading to cell proliferation in both types of cells. LDHB may control lysosomal acidification, vesicle maturation, and intracellular proteolysis in both oxidative and glycolytic type cancer cells [60]. LDHB activity generates protons (H^+^) in the process of conversion of lactate and NAD^+^ to pyruvate and NADH. The generated protons (H^+^) promote lysosomal acidification and autophagy in cancer. One of the post-translational mechanisms of LDHB regulation in cancer cell autophagy is via binding with protein Sirtuin 5 (SIRT5). SIRT5 a binding partner of LDHB, promotes LDHB enzymatic activity by deacetylating LDHB at lysine-329 position leading to increased autophagy and accelerated growth of cancer cells. SIRT5-induced *LDHB* deacetylation hyperactivates autophagy and targeting SIRT5/LDHB pathway could be highly useful in LDHB positive cancers [61]. 

Ribosomal protein S7 (RPS7) inhibits tumor growth by controlling glycolysis via suppressed expression of HIF-1α and LDHB. Increase in HIF-1α expression abolishes overexpression effects of RPS7 [62]. *LDHB* suppression also causes mitochondrial respiratory defects and plays role in cancer cell invasiveness by inducing the tight junction protein claudin-1 (CLN-1) [63]. A tissue microarray of stage I-III colorectal cancer (CRC) patient samples revealed that Krüppel-like transcription factor 14 (KLF14) expression was downregulated in CRC samples and the low KLF14 expression correlated with advanced tumor stage and size. Restoring KLF14 expression in vitro decreased rate of glycolysis significantly by downregulating *LDHB* [64]. Interestingly, higher *LDHB* expression in lung adenocarcinomas patients is a significant predictor of shorter survival and correlate with KRAS genomic copy number gain and mutation in both lung cancer cell lines and adenocarcinomas. The tumors with KRAS-mutation showed elevated glycolytic gene expression profile and depended more on glycolysis for proliferation as compared to KRAS wild-type lung tumors [65]. Table 2 shows some of mechanisms behind *LDHB* mediated tumor growth.

## 5. LDH-Mediated Metabolic Exchange between Cancer Cells and Stromal CAFs

The development of cancer is a complex multistep process; it occurs when a cell acquires six of the primary “cancer hallmarks” over time. The transformed cell acquires aberrant biological properties associated with sustained proliferative signaling, resist cell death by evading growth suppressors and develop replicative immortality. Abnormal proliferation of cancer cells results in faster consumption of available nutrients and oxygen results in a hypoxic, nutritionally stressed phenotype cancer. Consequently, the metabolic genes signal the cancer to reprogram itself in the stressed TME. One of the ways through which cancer cells rewire their metabolism, survive and adapt is by developing metabolic flexibility. Cross-talk with stromal cells helps cancer cells to satisfy their metabolic demands. Cancer cells also induce angiogenesis and activate invasion and metastasis to promote progression [66]. Attaining primary hallmark properties sets the foundation for cancer development and expedites the acquisition of additional secondary hallmark abilities [67] such as reprogramming energy metabolism [68] and evasion of immune surveillance [69]. One of the ways by which cancer cell achieve this is by recruiting a repertoire of apparently normal cells that create the “tumor microenvironment” [70]. Contrary to previous idea of tumors as specific homogeneous mass of proliferating cells the concept of tumor microenvironment revealed that tumors were actually a mass of complex tissues, composed of multiple distinct cell types. These apparently normal cells or stromal cells are not passive bystanders but play an active role in cancer development by sharing heterotypic interactions [71]. Stromal cells present in the malignant cancers get activated, become reactive and transition themselves into tumor-associated stromal cells (TASCs) helping to modulate the cancer phenotype. [72]. Overall metabolic reprogramming in the TME supports malignant cell growth. TASCs support cancer cells by providing additional nutrients as paracrine factors and supplement nutrient stock provided by the local vasculature [73].

In the tumor microenvironment, cancer-associated fibroblasts (CAFs) are one of the most heterogeneous and highly abundant stromal cell types, likely of mesenchymal origin. These are the dominant cell type with mesenchymal-like features present alongside neoplastic cells in solid tumors [72,74,75]. Multiple studies have highlighted the role of fibroblasts in TME and has given strength to argument that fibroblasts play a major role in supporting selfish tumors cells. Glycolytic tumor cells convert glucose to pyruvate and generate lactate. Generated lactate then exits the tumor cell by increased expression of lactate transporter MCT4. Increased concentration of lactate in the TME triggers *MCT1, LDHB* expression in the neighboring stromal cells such as hMSCs/CAFs which then take up the tumor-extruded lactate. The influxed lactate is converted to pyruvate with the help of LDHB present in CAFs. The generated pyruvate then fulfills the energetic demands of the CAFs [76] and is also shared with tumor cells through a reciprocally-supportive metabolic relationship [77]. Cross talk between cancer cells and fibroblasts also occur by another bi-directional interaction loop induced by reciprocal signaling of secreted components such as cytokines and other regulatory factors. This signaling makes positive feedback loops and promotes tumor growth [74]. Cancer mediated stromal metabolic reprogramming drives metabolic changes in the whole TME and provides metabolic resources by stromal-epithelial metabolic coupling [78]. Currently, almost all of the anticancer therapies target cancer cells specifically. Designing novel combination therapeutic strategies to block tumor stroma interaction may help in targeting cancers more effectively, particularly the stroma rich tumors [79]. Developing a better understanding of the molecular mechanism underlying signaling feedback loops may help in the development of novel molecular targeted therapies with improved efficacy. 

## 6. Lactate: A Substrate for LDH

**Figure 2:**
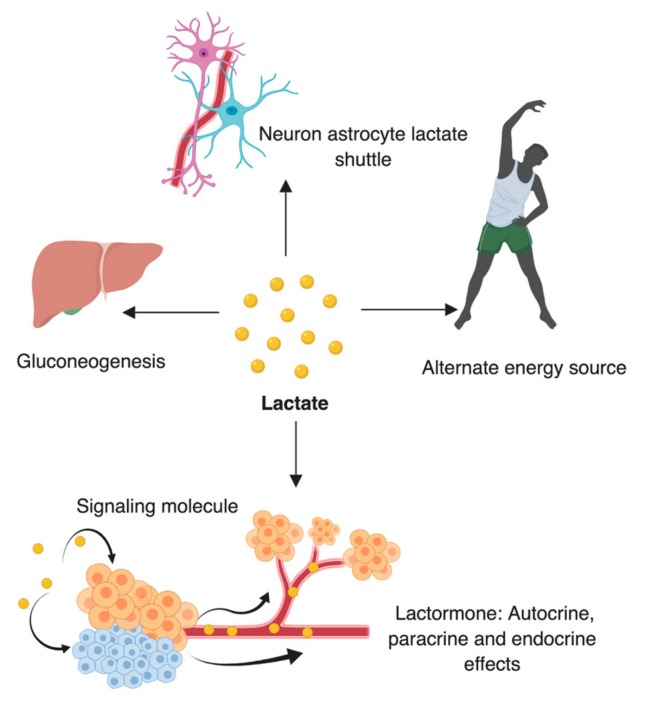
Physiological role of lactate in the body: Lactate acts as an alternate fuel in the body during endurance training; acts as energy source in brain through neuron astrocyte lactate shuttle; acts as a source for gluconeogenesis and also acts very often as a lactormone (hormone).

Lactate, a 3-carbon hydroxycarboxylic acid, previously considered as a waste product of anaerobic metabolism is continuously formed and utilized by different types of cells under fully aerobic conditions [80,81]. In addition, lactate shuttles between producer and consumer cells in the body and plays critical role in normal physiology of human body including, a major energy source, a major gluconeogenic precursor and a signaling molecule [81]; as shown in Figure 2. 

In the human body lactate is mainly synthesized from glucose and alanine and is predominantly consumed and released by skeletal muscles, heart and brain [80]. Under normal conditions, highest lactate levels are found in muscles and are cleared out of the body mainly by liver and in trace amount by kidneys [81,82]. At times lactate can serve as an alternate source of energy by being reconverted into glucose in the liver via the Cori cycle [83,84]. For complete lactate oxidation, lactate must be present in the cell, either by directly entering the cell or by being produced internally. Adequate oxygen concentration and healthy mitochondrion is also required for proper lactate oxidation [83]. Membrane-bound monocarboxylate transporter like MCT1 makes the in and out movement of lactate feasible and enzymes LDH makes the interconversion of lactate to pyruvate possible. MCT1 is mainly involved in lactate uptake, whereas, MCT4 is involved in lactate release [85]. 

**Figure 3:**
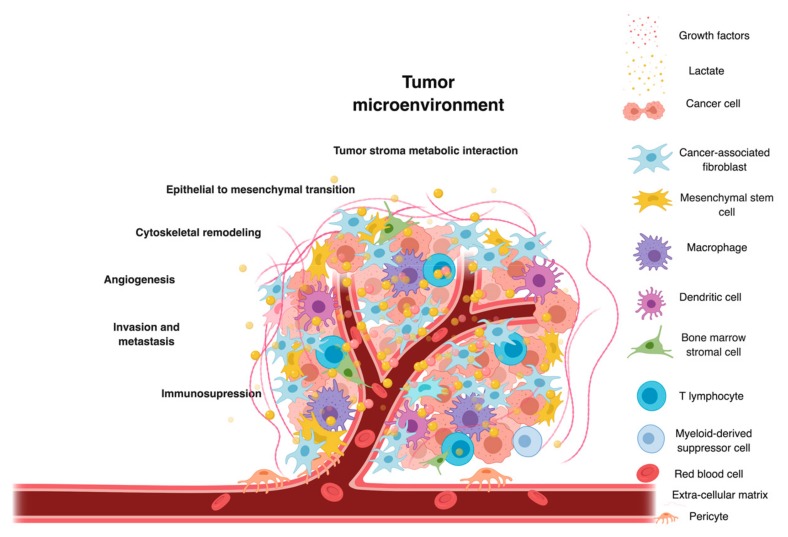
Role of lactate in the tumor microenvironment (TME): A collection of cancer cells with an army of recruited stromal cells, nutrients and growth factors. Metabolic interactions between cancer cells and stromal cells regulate the process of carcinogenesis. One of the primary metabolites through which cancer and stromal cells crosstalk is lactate. Lactate acts as a key player in cancer progression and activates epithelial to mesenchymal transition, cytoskeletal remodeling; promotes immunosuppression and angiogenesis, invasion and metastasis.

Cancer cells contain significant levels of lactate and recent finding have established clinical correlation between tumor lactate levels with higher metastasis, recurrence and poor treatment outcome [86]. As already discussed in previous sections lactate helps in promoting tumor growth in several ways as shown in Figure 3. It is now known that lactate present in the microenvironment can be metabolized as a secondary energy source by tumor cells and can be shuttled back to neighboring cancer cells, stromal cells, and vascular endothelial cells [87]. As initially observed by Warburg, a broad range of lactate-producing or ‘lactagenic’ cancer cells are characterized by increased rate of aerobic glycolysis and excessive lactate formation. These lactagenic cancers work in a highly organized manner to fulfill increased glucose demands for lactagenesis and follow different steps such as increasing glucose uptake and glycolytic enzyme expression, decreasing mitochondrial function, increasing overall lactate production, accumulation and release and finally upregulating expression of monocarboxylate transporters MCT1 and MCT4 for lactate exchange among different cells [84]. 

## 7. Role of Lactate in Regulating Immunometabolism in the TME

In a cellular microenvironment, immune cells and other cells face competition to share nutrients such as glucose, amino acids, fatty acids and metabolites such as lactate. A fine balance between these factors mandates proper functioning of T cells and regulate overall immune response and disease development [88]. Chang et al. have shown that increased glucose consumption by tumors restricts T cells metabolically and helps in tumor progression by weakening mTOR activity, glycolytic capacity, and production of IFN-γ [89]. Naive T cells depend initially on OXPHOS but later augment with glycolytic metabolism to fulfill their metabolic demands and this dependency on glycolytic metabolism separates T cells including CD4 Th1, Th2, and Th17 effector cells from T reg cells [90]. Lactate provides immuno-metabolic coupling between cancer cells and other stromal cells and helps in dampening immune-surveillance by impairing cytotoxic function of CD8+ T-cells [91], upregulating inhibitory molecules, downregulating costimulatory molecules, increasing production of immunosuppressive cytokines [92] inhibiting monocytes differentiation to dendritic cells (DC) and eventually inactivating cytokine release from DCs [93]. High-lactate levels in the TME helps in metabolic adaptations in several ways; one way is via Foxp3, a Treg transcription factor, that metabolically reprograms T cell by suppressing glycolysis and enhancing oxidative phosphorylation allowing Tregs to work efficiently in conditions of low-glucose and high lactate [94]. Macrophage activation is also associated with metabolic remodeling and metabolic reprogramming of macrophages mediated by mTORC2-IRF4 signaling axis, indispensable for their alternative activation [95]. Inflammatory pathway (NF-κB) also plays a major role in tumor stroma interaction [96] Lactate modulates tumor microenvironment by directly entering endothelial cells via MCT-1 and activating the phosphorylation/degradation of IκBα to stimulate the autocrine NF-κB/IL-8(CXCL8) pathway leading to cell migration, angiogenesis and tumor growth [97]. Changes in cellular metabolism and immune system drive cancer development and progression and effective therapeutic designing targeting these two areas in the TME would be beneficial [98]. These studies highlight the immuno-suppressive role of lactate in tumors and emphasize targeting immunometabolism in the TME for development of future therapies.

## 8. Serum LDH Profile in Cancers and Clinical Relevance

Lactate dehydrogenase present in cells is released into the bloodstream when the cell is damaged and is measured by LDH serum profiling. Serum LDH levels can be of great significance as prognostic marker in various solid cancers such as colorectal cancer [99], lung cancer [100], nasopharyngeal cancer [101], prostate cancer [102] and breast cancer [103] patients among others. Meta-analysis study on patients with advanced disease showed highly significant association between elevated serum LDH levels and poor survival in patients with solid tumors such as renal cell carcinoma, prostate, gastric, melanoma, nasopharyngeal and lung cancers and highlights use of LDH as a prognostic biomarker in advanced carcinomas [104]. Meta-analysis studies on colorectal [105] and urologic cancer [106] have also shown association of LDH levels with poor overall survival. Pretreatment serum LDH level along with TNM staging might more accurately predict disease risk definition and can serve as an independent, robust, reliable biomarker for predicting overall survival (OS), disease-free survival (DFS) as well as distant metastasis-free survival (DMFS) [107]. Serum LDH is also an independent predictor of decreased progression-free survival (PFS) in thymic carcinoma patients [108] as well as in high-grade osteosarcoma patients where it has been found to be a strong predictor of skeletal metastasis [109]. Hepatocellular carcinoma (HCC), patients with higher preoperative serum LDH levels have a worse prognosis following hepatectomy for both OS and DFS [110]; Ewing′s sarcoma patients with similarly high levels of preoperative serum LDH showed lower OS and 5-year DFS rates [111]. 

Apart from being a significant prognostic predictor of OS, serum LDH also serve as predictors of response to chemotherapy. Serum LDH levels, in advanced pancreatic cancer patients treated with gemcitabine-based palliative chemotherapy were found to be a predictor of OS and were associated with the systemic inflammatory response [112]. Serum LDH levels were independent, unfavorable prognostic predictor for OS in nasopharyngeal carcinoma (NPC) patients treated with neoadjuvant chemotherapy (NCT) and conventional radiotherapy (RT) or concomitant chemoradiotherapy (CCRT) [101]. Advanced triple-negative breast cancer (TNBC) patients (age > 40 years) showed significantly elevated baseline LDH levels (>250 IU/L) and a strong association was found between serum LDH levels, high number of metastatic sites and poor prognosis. The progression-free survival PFS was reduced in patients with higher post-treatment LDH levels and predicted poor OS and poor response to first-line chemotherapy [113]. 

## 9. Future Directions in Therapeutic Designing

As discussed previously the differential expression of LDHA and LDHB has been reported in multiple malignancies and is often clinically correlated with disease outcome. It would be of great advantage to target LDHA and LDHB for future therapeutic designing. Some of the possible ways of effective targeting are the use of small molecule inhibitors and small interfering RNA mediated molecular inhibition. 

### 9.1. Small Molecule Inhibitors

In this regard in silico and in vitro studies done in the last few years have revealed *LDHA* and *LDHB* as potential therapeutic targets. Identification of selective, small molecule inhibitors as lead candidates for LDH mediated cancer targeting holds promise [114] and their combined use with specific pathway inhibitors will help in broadening clinical utility in tumors of different metabolic types [115]. To develop small molecule inhibitors, different in silico approaches such as fragment-based lead generation (FBLG) along with assisted by X-ray crystallography [116], molecular dynamics (MD) and simulations [117,118], receptor-based pharmacophore modeling approach [119], structure-based virtual screening [120] have been used. Preclinical in vitro studies done with small molecules inhibitors of both natural and synthetic origin have shown potential in blocking LDH expression. 

FX11 (3-dihydroxy-6-methyl-7-(phenylmethyl)-4-propylnaphthalene-1-carboxylic acid) is a NADH competitive, selective, small molecule inhibitor of LDHA that was reported to inhibit cancer progression in multiple cancer [121]. In gallbladder carcinoma (GBC) specific inhibitor FX11 mediated LDHA silencing significantly suppressed cancer cell proliferation, invasion, growth and induced GBC cell apoptosis [122]. Inhibition of overexpressed LDHA in prostate cancer cells by FX11 inhibited cell proliferation, migration, invasion and induced apoptosis [123]. Gossypol, a natural phenolic product derived from cotton seed has also been reported to inhibit tumor growth [124,125]. It has shown dose dependent cytotoxicity in different cancers cells such as melanoma, small cell lung cancer, breast cancer, cervical cancer, myelogenous leukemia and glioma [126,127]. Gossypol has also shown satisfactory anticancer activity in metastatic breast cancer [128], metastatic adrenal cancer [129] and gliomas [130] Galloflavin is another artificially synthesized inhibitor found to inhibit both LDHA and LDHB [131]. Galloflavin preferentially binds to free enzymes and does not competes with substrate and cofactors [132] and has shown anticancer activity in human breast cancer cells [133].

N-Hydroxyindole-based inhibitors are competitive, small molecule inhibitors that compete with pyruvate and NADH (126) and display good anti-proliferative and starvation inducing abilities in cancer [134,135,136,137]. Oxamate sodium, an analogue of pyruvate, also inhibits tumor growth by attenuating glycolysis [31,32]. 

### 9.2. Molecular Inhibition of LDHA and LDHB

Small interfering RNAs (siRNAs) are 20–25 nucleotides long, double-stranded RNA molecules that downregulate gene expression by targeting sequence complementarity. siRNA mediated gene expression inhibition has become a major tool in cancer research and offers promising future avenues [138]. Use of siRNAs in targeting LDHA have been shown by several groups as an effective way to stop cancer progression [139,140,141,142,143,144]. siRNA-mediated knockdown of overexpressed *LDHA* in renal carcinoma cells inhibited cell proliferation and induced apoptosis by targeting proteins such as Bcl-2, Bax, p21, cyclin D1 and also reduced matrix metalloproteinase (MMP)-2 and MMP-9 expression [139]. Similarly, in glioblastoma, knockdown of *LDHA* expression decreased cell growth, reduced glycolysis and induced increased apoptosis [140]. Knockdown of *LDHA* overexpression in colorectal cancer cells inhibited growth rate and reduced lactate and ATP production as well as glucose uptake [141]. shRNA mediated knockdown of *LDHA* increased mitochondrial ROS production and decreased cell proliferation and motility in MDA-MB-435 cancer cell line and was found to be associated with cytoskeletal remodeling [30]. Lentiviral vector mediated RNA interference (RNAi) of LDHA in hepatocellular carcinoma (HCC) cells increased apoptosis by increasing reactive oxygen species production and significantly reduced metastatic potential in a xenograft mouse model [142]. Depletion of *LDHA* expression by CRISPR/Cas9 and shRNA in neuroblastoma cells inhibited tumor growth, clonogenicity and tumorigenicity without abolishing LDH activity or significantly reducing aerobic glycolysis [143]. Knocking down of HIF1/2α activated *LDHA* expression in human pancreatic cancer cells, decreased cell growth and migration [144]. Inhibiting LDHA has also been shown to make cells more sensitive to radiation and chemotherapy. siRNA mediated knockdown of *LDHA* expression in Glioblastoma multiforme arrested cell growth by blocking cell cycle progression and inducing apoptosis in these cells and increased chemo sensitivity to temozolomide [145]. shRNA mediated *LDHA* attenuation also stimulates mitochondrial respiration and is shown to cause a decrease in mitochondrial membrane potential and a compromised ability to proliferate under hypoxia [146]. *LDHA* inhibition by siRNA or FX11 also leads to induction of oxidative stress and cell death, which could be an effective treatment strategy for LDHA-dependent tumors [121]. *LDHA* expression in prostate cancer cells has been associated with radio resistance and siRNA mediated knock down re-sensitized cells to radiotherapy, decreased epithelial-mesenchymal transition and increased apoptosis [147]. In malignant cartilage-forming chondrosarcoma inhibiting LDHA increased cancer cell sensitivity to doxorubicin [148] and in breast cancer cells led to re-sensitization to Taxol (paclitaxel) [149]. Inhibition of *LDHA* and *LDHB* expression by small molecule inhibitors or by non-coding RNA approach would be of great interest and could possibly interfere with cancer progression. Future work in this area is ongoing and results are awaited. Inhibition of *LDHA* and *LDHB* is unlikely to cause any possible side effect, Therefore, it could be highly beneficial to look for novel inhibitors as complimentary chemotherapeutic agents and treatment sensitizers. 

### 9.3. Blocking Lactate Exchange between Tumor and Stroma

CAFs act selflessly in a subservient manner to cancer cells by offering available glucose to them and utilizing lactate secreted by their masters. It may be useful to target LDHA on cancer cells and LDHB on stromal cells for breaking the reciprocal exchange of nutrients between tumor and stromal cells, inhibiting tumor proliferation. Giatromanolaki et al. have done metabolic interactions analysis between stromal and epithelial elements and shown that prostate cancer cells mainly express LDH-5 whereas the tumor-associated fibroblasts/myofibroblasts (TAFs) express LDH-1. They have highlighted on the fact that both of the isoenzyme acts complementary. The LDH-5 isoenzyme present on cancer cells converts pyruvate to lactate, whereas the LDH-1 isoenzyme present on CAFs/TAS utilizes the secreted lactate and converts it to pyruvate, essential source of energy for cancer cells [150]. Lactate produced in the TME plays multiple critical roles in promoting various aspects of metabolic regulation [151,152] and acts as a metabolic driver of cancer landscape [153]. Patel et al. have also shown that stromal CAFs recycle tumor secreted lactate for meeting their own energetic requirements and overall spare glucose for neighboring glycolytic tumor cells [76,77]. Experimental data on *LDHB* knockout mice generated by our group has further strengthened the role of stromal LDHB in supporting tumor growth (manuscript in preparation). Hence, finding ways to block lactate exchange between tumor and stroma would be of benefit.

## 10. Conclusions

Cancer is a continuously evolving disease with abnormal bioenergetic metabolism. Cancer cells have the ability to reprogram metabolic pathway for fulfilling elevated nutrients demands to support a high rate of proliferation. As reported in different types of cancers glycolytic pathway is often deregulated to meet the accelerated bioenergetic and metabolic demand. Cancer cells frequently reprogram their own metabolic pathways as well as those of neighboring stromal cells. LDH is one of the primary enzymes that link tumor and stroma. One of the ways through which LDHs help in tumor progression is by tumor stroma interaction. Carcinoma associated fibroblasts (CAFs) are one of the most important stromal cell types present in the tumor microenvironment. Other than being a fuel for CAFs, the lactate generated by cancer cells in the tumor microenvironment helps in tumor progression by extracellular matrix remodeling, directly and indirectly activating signaling pathways and releasing inflammatory molecules and dampening immune responses. As deregulated levels of *LDHA* and *LDHB* are associated with poor prognosis and inferior therapeutic outcome; blocking lactate flux inside the TME may serve as a novel therapeutic target and could help in designing future complimentary therapies. Immunocompetent animal models to study the effect of small molecule inhibitors for LDH inhibition will help in better understanding of molecular interaction between tumor and stroma with respect to immune modulation leading to rational drug development. Further, heterogeneity in LDH expression among different cancers warrants more cell type specific research in this area for effective therapeutic designing.

## Figures and Tables

**Table 1:** *LDHA*-mediated cancer progression.

Cancer Type	Sample/Model Systems	Mechanisms	Expression	Reference
Pancreatic cancer	pancreatic tumors and cancer cell lines	increased pancreatic cancer cell growth and metastasis by FOXM1-LDHA signaling	*LDHA* overexpression	[8]
Pancreatic cancer	pancreatic tumors, in vitro and in orthotopic mouse models	increased pancreatic cancer cell growth and metastasis by KLF4-LDHA signaling	*LDHA* overexpression	[9]
Pancreatic cancer	pancreatic ductal carcinoma samples	increased pancreatic cancer cell growth by citrate synthase mediated glucose to lipid conversion	*LDHA* overexpression	[10]
Nasopharyngeal cancer	NPC tumor tissues and cell lines	increased cell proliferation, migration and invasion by JMJD2A-LDHA signaling	*LDHA* overexpression	[12]
Gastric cancer	GC cell lines	promoted cancer growth by glycolytic phenotype (increased lactate production and glucose utilization) by FOXM1-LDHA signaling	*LDHA* overexpression	[13]
Bladder cancer	invasive transitional cell bladder cancer tissues	poor local relapse free survival, LDHA Silencing lead to strong radio-sensitization.	LDH-5 over expression	[14]
Endometrial cancer	stage I endometrial adenocarcinoma cells	LDH-5 mediated expression of phosphorylated VEGFR2/KDR receptors pathway	LDH-5 over expression	[15]
Bladder cancer	BC cell lines, muscle-invasive bladder cancer samples	epithelial-to-mesenchymal transition	*LDHA* expression	[31]

**Table 2:** *LDHB*-mediated cancer progression.

Cancer Type	Sample/Model System	Mechanism	Expression	Reference
Prostate cancer	poorly metastatic and highly metastatic variant of human prostate cancer cell lines and primary cancer tissues	promoter hypermethylation	loss of *LDHB* expression	[51]
Gastric cancer cell lines and pancreatic cancer	four gastric cancer cell lines and one pancreatic cancer cell line	promoter hypermethylation	loss of *LDHB* expression, loss of LDH1-4	[52]
Pancreatic cancer	pancreatic cancer tissues and cell lines	promoter hypermethylation	suppressed *LDHB* and even total loss	[53]
Breast cancer	breast cancer (adenocarcinoma) tissues and cell lines	promoter hypermethylation	Absent or decreased expression of LDH isoenzymes 1–4	[54]
Breast cancer	basal-like/triple-negative breast cancers	glycolytic pathway	high *LDHB* expression	[55]
Hepatocellular carcinoma	Highly metastatic cell line	Metastasis potential	down-regulated LDHB expression	[56]
Hepatocellular carcinoma	HCC cancer tissues	unfavorable survival outcomes	Low *LDHB* expression	[57]
Urinary bladder urothelial carcinoma (UBUC)	UBUC cancer tissues	tumor progression and inferior disease-specific survival	Loss of *LDHB* expression	[58]
Colorectal cancer	colorectal cancer tissues, in vitro and in vivo model	increased autophagy, accelerated cancer growth mediated by SIRT5/LDHB pathway	Deacetylation of LDHB	[61]
Colorectal cancer	colorectal cancer tissues, CRC cell lines,	RPS7 mediated cell glycolysis inhibition, inhibits colorectal cancer growth	Suppressed *LDHB* expression	[62]
Hepatoma cancer	hepatoma cell lines	claudin-1-mediated high invasive activity	Decreased *LDHB* expression	[63]
Colorectal cancer	stage I-III CRC patient samples, in vitro	KLF14 regulates glycolysis	downregulating *LDHB* expression	[64]
Lung cancer	KRAS-dependent lung adenocarcinoma samples, vitro and in vivo	predictor of shorter survival in patients, KRAS-mutant lung tumors are more dependent on glycolysis for proliferation compared with KRAS wild-type lung tumors	high *LDHB* expression	[65]
