# Correction: Mishra, et al.; Lactate Dehydrogenases as Metabolic Links between Tumor and Stroma in the Tumor Microenvironment. *Cancers* 2019, *11*, 750

**DOI:** 10.3390/cancers12040932

**Published:** 2020-04-09

**Authors:** Deepshikha Mishra, Debabrata Banerjee

**Affiliations:** Department of Pharmacology, Robert Wood Johnson Medical School, Rutgers, The State University of New Jersey, 675 Hoes Lane West, Research Towers, Room 561, Piscataway, NJ 08854, USA

The authors would like to make a correction to their published paper [1].

The authors would like to change one incorrect sentence in [1].

On page 3, in paragraph one, the sentence: “A difference in net charge on the molecule guides choice of ligand with higher affinity; LDHA with a negative 6 (−6) net charge has higher affinity for pyruvate as compared to a net positive charge (+1) of LDHB which has higher affinity for lactate [24].” should be changed to “A difference in net charge on the molecule guides the choice of the ligand with higher affinity; LDHB with a negative 6 (−6) net charge has a higher affinity for lactate as compared to a net positive charge (+1) of LDHA, which has a higher affinity for pyruvate [24].”

The change does not affect the scientific results.

The rest of the manuscript does not need to be changed. The authors would like to apologize for any inconvenience caused. The manuscript will be updated, and the original will remain available on the article webpage.
